# Supplementary material for: Developing a national birth cohort for child health research using a hospital admissions database in England: The impact of changes to data collection practices
Source: PLoS One. 2020 Dec 15;15(12):e0243843. doi: 10.1371/journal.pone.0243843 (PMC7737962; doi:10.1371/journal.pone.0243843)
Supplement: S1 Appendix — (DOCX) [file pone.0243843.s001.docx]

# S1 Appendix – Hospital Episode Statistics (HES) linkage algorithms

S1 Table A – Algorithm for generating the HESID for linkage of hospital admissions within HES. A set of records are allocated the same HESID if a match is found using any of the three rules below. If no match is found, a new HESID is generated.(1)

| **Rule No.** | **NHS Number** | **Date of birth** | **Sex** | **Postcode** | **Other** |
| --- | --- | --- | --- | --- | --- |
| 1. | Exact match | Partial match | Exact match | - | - |
| 2. | Not conflicting (e.g. missing in one record) | Partial match | Exact match | Exact match | Exact match on local patient identifier within a hospital and hospital code |
| 3. | Not conflicting (e.g. missing in one record) | Exact match | Exact match | Exact match | - |

HES=Hospital Episode Statistics. NHS = National Health Service

S1 Table B – Algorithm for generating the HESID for linkage of ONS mortality records data with HES hospital admission records. A death record is allocated a HESID, according to the eight rules below. If multiple matches occur, the highest quality of a match is retained.(2)

| **Match rank** | **NHS Number** | **Date of birth** | **Sex** | **Postcode** |
| --- | --- | --- | --- | --- |
| 1: highest quality match | Exact match | Exact match | Exact match | Partial match |
| 2 | Exact match | Exact match | Exact match | - |
| 3 | Exact match | Partial match | Exact match | Partial match |
| 4 | Exact match | Partial match | Exact match | - |
| 5 | Exact match | - | - | Exact match |
| 6 | Not conflicting (e.g. missing in one record) | Exact match **& not 1st January** | Exact match | Exact match  **& and not a communal establishment (e.g.: hospital, prison, army barracks, etc.)** |
| 7 | Not conflicting (e.g. missing in one record) | Exact match **& not 1st January** | Exact match | Exact match |
| 8: lowest quality match | - | Exact match **& not 1st January** | Exact match | Exact match |

HES=Hospital Episode Statistics, NHS = National Health Service, ONS = Office for National Statistics

**References:**

1. The Health and Social Care Information Centre (HSCIC). Methodology for creation of the HES Patient ID (HESID) [Internet]. 2014. Available from: https://webarchive.nationalarchives.gov.uk/20180328130852tf_/http://content.digital.nhs.uk/media/1370/HES-Hospital-Episode-Statistics-Replacement-of-the-HES-patient-ID/pdf/HESID_Methodology.pdf/

2. The Health and Social Care Information Centre (HSCIC). A Guide to Linked Mortality Data from Hospital Episode Statistics and the Office for National Statistics. 2015;(June). Available from: https://digital.nhs.uk/binaries/content/assets/legacy/pdf/r/q/hes-ons_linked_mortality_data_guide.pdf
