# Supplementary material for: Developing a national birth cohort for child health research using a hospital admissions database in England: The impact of changes to data collection practices
Source: PLoS One. 2020 Dec 15;15(12):e0243843. doi: 10.1371/journal.pone.0243843 (PMC7737962; doi:10.1371/journal.pone.0243843)
Supplement: S2 Appendix — (DOCX) [file pone.0243843.s002.docx]

# S2 Appendix – Developing a cohort of singleton live births in HES

Our Stata code used to derive the cohort is available on GitHub repository: <https://github.com/UCL-CHIG/HES-birth-cohorts>. We first extracted data for all hospital admissions in the first year of life (where age at start of the episode was <1 year old, in HES indicated as *startage>7000*) and infant deaths from the HES-ONS mortality dataset. We applied very basic data cleaning to both extracts to remove implausible values, ensure consistent coding of missing values etc (see do-files “*1. HES - basic cleaning for recorded variables.do*” and “*2. HES-ONS death file - basic cleaning for recorded variables.do*”).

## Developing a birth cohort: inclusion and exclusion criteria

To develop a birth cohort, we extracted all HES episodes with an age at start of episode <7 days (i.e. *startage*<7003 in HES) and linked HES-ONS mortality records. We then applied broad selection criteria based on diagnostic and procedure codes, healthcare resource group codes and administrative variables recorded in HES (such as admission method or level of provided neonatal care) to identify birth episodes. All inclusion criteria are listed in S2 Table A. We excluded multiple births, terminations of pregnancy, stillbirths. Exclusion criteria are detailed in S2 Table B.

Next, we carried out additional data cleaning to ensure we derive one birth episode per HESID. We excluded unfinished episodes (using *epistat* variable in HES, see S2 Table B) as they should not contain any clinical details and more complete episodes should be recorded within HES. We cleaned and validated date variables (such as admission and discharge dates) which can contain recording errors (e.g., if recorded admission date was after discharge). Finally, we de-duplicated the episodes (these steps are also detailed in S2 Table B).

Next, we removed implausible values of gestational age (we replaced gestational age to missing if gestational age was <22 or >45 weeks, *gestat* variable in HES), birth weight (replaced to missing if <200g, *birweit* variable in HES) and maternal age (replaced to missing if <10 or >60*, matage* variable in HES). We copied information about these variables across all episodes of care with age at admission <7 days. We indicated and dropped HESIDs for which information for birth weight, gestational age, maternal age or month and year of birth differed between multiple episodes of care as we assumed they were likely to be false matches, i.e. records for different children who were allocated the same HESID. We then kept one row per HESID and saved only relevant variables recorded at birth (birth weight, gestational age, maternal age, sex).

Lastly, we replaced implausible combinations of birth weight and gestational age as missing. These were indicated if the recorded birth weight fell outside +/-4 standard deviations (SD) of mean birth weight for each gestational age. To obtain birth weight centiles, we used LMSgrowth, a Microsoft Excel add-in with growth references for children in the UK, developed by Pan and Cole.(1) For preterm babies, we used birth weight centiles based on the *UK WHO preterm* reference in LMSgrowth, which was extrapolated to 22 weeks; for term babies born from 37 to 42 weeks we used *UK WHO term* reference and we used the values for 42 weeks as cut-offs for higher gestations. These centiles are sex-specific. If sex was missing, we used the overlapping values between the two sexes as cut-off points.

All of these steps are implemented in “*3. Deriving a birth cohort in HES data.do*” do-file. At this stage, we have not yet removed non-English residents from the cohort due to a postcode extraction error for episodes indicated as births. This exclusion criterion was applied after we cleaned hospital admission and mortality records (details are described in sections 2 and 3 of this document), when finalising the cohort (details described in section 4).

S2 Table A – Criteria for identifying birth episodes in HES

| Variable used | | Inclusion Criteria  (value recorded in HES and explanation) |
| --- | --- | --- |
| Diagnostic codes  (ICD-10) | | Z38: Liveborn infants according to place of birth and type of delivery  Z37: Outcome of delivery |
| Healthcare Resource Group Codes | **version 3.5** | N01: Neonates - Died <2 days old  N02: Neonates with Multiple Minor Diagnoses  N03: Neonates with one Minor Diagnosis  N04: Neonates with Multiple Major Diagnoses  N05: Neonates with one Major Diagnosis |
|  | **version 4.0**  **(in use since financial year 2011/12)** | PB01Z: Major Neonatal Diagnoses  PB02Z: Minor Neonatal Diagnoses  PB03Z: Healthy Baby |
| HES Specific Fields | **Episode type** *(epitype)* | 3: Birth episode  6: Other birth event |
|  | **Patient classification**  *(classpat)* | 5: Mothers and babies using only delivery facilities |
|  | **Admission method**  *(admimeth)* | 82: Other: babies born in health care provider  83: Other: babies born outside the health care provider, except when born at home as intended  2C: Baby born at home as intended (available from 2013/14) |
|  | **Neonatal Care**  *(neocare)* | 0: Normal care  1: Special care  2: Level 2 intensive care (high dependency intensive care  3: Level 1 intensive care (maximal intensive care) |

HES, Hospital Episode Statistics; ICD-10, International Statistical Classification of Diseases and Related Health Problems. Financial years in England run from 1st April to 31st March the following year.

S2 Table B – Exclusion criteria for multiple births, stillbirths and terminations of pregnancy

|  | Variable used | | Exclusion criteria (value recorded in HES and explanation) |
| --- | --- | --- | --- |
| **Multiple Births** |  | Diagnostic codes  (ICD-10) | Z372: Twins, both liveborn  Z373: Twins, one liveborn and one stillborn  Z374: Twins, both stillborn  Z375: Other multiple births, all liveborn  Z376: Other multiple births, some liveborn  Z377: Other multiple births, all stillborn  Z383: Twin, born in hospital  Z384: Twin, born outside hospital  Z385: Twin, unspecified as to place of birth  Z386: Other multiple, born in hospital  Z387: Other multiple, born outside hospital  Z388: Other multiple, unspecified as to place of birth |
|  | HES Specific Fields | Birth order  (*birordr*) | greater than 1 (*birordr*>1) |
|  |  | Number of babies (*numbaby*) | more than 1 (*numbaby*>1) |
| **Termination of pregnancy** | | Diagnostic codes  (ICD-10) | P964: Termination of pregnancy, affecting fetus and newborn |
| **Stillbirth*** |  | Diagnostic codes  (ICD-10) | P95: Fetal death of unspecified cause  Z371: Single stillbirth  Z373: Twins, one liveborn and one stillborn  Z374: Twins, both stillborn  Z376: Other multiple births, some liveborn  Z377: Other multiple births, all stillborn |
|  | HES Specific Fields | Discharge method (*dismeth*) | 5: Baby was still born |
|  |  | Birth status  (*birstat*) | 2: Still birth: ante-partum  3: Still birth: intra-partum  4: Still birth: indeterminate |

HES, Hospital Episode Statistics; ICD-10, International Statistical Classification of Diseases and Related Health Problems. Financial years in England run from 1st April to 31st March the following year. *We assumed that stillbirths that linked to a death record with a high certainty (indicated by a match rank of 1 or 2) were live births miscoded as stillbirths and we kept them in the cohort.

## Cleaning and linking hospital admissions in the first year of life

We extracted all episodes of care in the first year of life for babies identified in the HES birth cohort. We removed episodes with no clinical information recorded (e.g., unfinished episodes). We then cleaned and validated date variables (such as admission and discharge dates) which can contain recording errors (e.g., if the recorded admission date was after the discharge date). Finally, we de-duplicated the episodes. Details of data cleaning rules are described in S2 Table C.

We then linked episodes into admissions using an algorithm developed by Hardelid et al.(2) An admission was defined as a continuous period of time that a child spent under NHS hospital care. Hospital transfers and admissions within 1 day of each other were treated as one inpatient admission. Lastly, we derived the most commonly recorded value of region of residence, IMD score and post code per admission. All of these steps are presented in “*4 - HES - cleaning and linking hospital admissions in infancy.do*” do-file.

S2 Table C – Cleaning rules for HES longitudinal records

| Criterion | | | | Action | |
| --- | --- | --- | --- | --- | --- |
| Drop episodes with no clinical information recorded | | Unfinished episodes | | Drop (as usually more complete record was available) | |
|  |  | Missing episode end date | | Drop (as usually more complete record was available) | |
|  |  | Only clinical information recorded was diagnosis “R69” – “*Illness, unspecified*” | | Drop (as usually more complete record was available) | |
|  |  | No recorded diagnoses | | Drop (as usually more complete record was available) | |
| Validate and correct date variables (admission and discharge dates, episode start and end dates) | | Admission date missing | | Replace to episode start date for the first episode of the admission (*epiorder*=1)  Else, replace to admission date from episode with episode order smaller by 1 and closest episode start date  Else, replace with episode start date | |
|  |  | Episode start date missing | | Replace to admission date for the first episode of the admission (*epiorder*=1)  Else, replace to episode end date from another episode with the same admission date and lower episode order | |
|  |  | Episode end date missing | | Removed as part of exclusion criteria | |
|  |  | Episode start> episode end | | Replace episode start with admission date if the issue is with the recording of episode start (episode start > episode end ≥ admission date)  Replace episode end with episode start date if the issue is with the recording of episode end (episode start = admission date > episode end)  Switch episode start with episode end, and admission date with discharge date if they were incorrectly recorded (episode start > episode end & admission date > discharge date where discharge date is not missing) | |
|  |  | Admission date > episode start | | Replace episode start date with admission date | |
|  |  | Admission date > episode end | | Replace episode end date with episode start date | |
| Validate and correct date variables (admission and discharge dates, episode start and end dates) | Discharge date missing | | Discharge date is recorded only on the last episode of care. Therefore, I generated a maximum discharge date by HESID and admission date as the “complete” discharge date.  If “complete” discharge date was missing (when discharge date was not recorded for an admission), I replaced it with maximum episode end date by HESID and admission date.  If “complete” discharge date was smaller than the maximum episode end date, I replaced it with the maximum episode end date. | |  |
|  | Episode ends in a different year than it starts | | Drop if the difference is greater than or equal to two. It seems impossible to be seen by only one consultant while staying in the hospital for 2 years so it must be a data error. | |  |
|  | Missing episode start age | | No such observations | |  |
|  | Missing episode end date | | Generate an age using episode start and end dates for episodes with startage=7001 (“less than 1 day”) | |  |
|  | Age at start of episode > age at end of episode | | Switch age at start with age at end of episode | |  |
|  | Epistart – Epiend > 365 | | Episodes that lasted more than 1 year were assumed to be recording errors and dropped as it is unlikely that a patient would be seen by just one consultant for that long. | |  |
| Drop duplicates | Exact duplicates | | Drop duplicates in terms of: HESID, age at start and end of admission, month and year of birth, gender, post code, start and end date of the episode, episode order, admission and discharge dates, provider code,all diagnoses and operations and cause of injury | |  |

HES, Hospital Episode Statistics; IMD, Index of Multiple Deprivation.

## Cleaning and linking ONS mortality records

The ONS mortality data was linked to births identified in HES using the HESIDs provided by NHS Digital. We identified additional deaths not indicated by NHS Digital by flagging hospital admission records where the discharge method indicated death. We included only deaths in the first year of life which occurred before 31^st^ December 2016. We also excluded likely false matches, which we defined as:

- records with a date of death before the estimated birth date:
  where the difference between the two dates was greater than one day, or if there was any difference between these dates for links with the poorest quality match rank (that is, where the NHS number was not required to match – match rank 8 in S1 Appendix Table B)
- records with subsequent hospital admissions after death:
  where there was >1 day’s difference between the last admission date and death date (admissions one day after death could occur if for example, test results are released and recorded in the system after discharge and death)(3)
- records for in-hospital deaths where the difference in the date of discharge (and death) in admission record in HES and the date of death in ONS mortality records was >1 day:
  a difference of one day was deemed to be plausible if, for example, discharge was not possible on the day of death or some test results emerged after the death of a patient(3)

All of these steps are described in “*5. HES-ONS death file - further cleaning and linkage validation.do*” do-file on GitHub.

## Finalising the birth cohort: additional data cleaning

To finalise the birth cohort we have linked in additional variables derived from linked hospital admission and mortality records, and we excluded non-English residents. Details are described in “*6. finalising HES birth cohort.do”* do-file on GitHub.

### Ethnicity, IMD score

Ethnicity, county of residence and index of multiple deprivation (IMD) score (variables *ethnos, rescty, imd04rk* in HES, respectively) were completed using longitudinal hospital admission records. Ethnicity was completed using the most commonly recorded value across all hospital admission records. County of residence and IMD score were completed using the earliest recording in any hospital admission record the first year of life.

### Length of birth admission

We derived the length of birth admissions as the difference between discharge and admission date for the first admission in cleaned follow-up hospital admission records.

### Excluding non-English residents

We used county of residency variable (*rescty* in HES) to indicate babies born to non-resident mothers. Since this variable was more likely to be missing in HES, we used the earliest recording in any hospital admission record the first year of life. Babies were excluded if their mothers were indicated to be residents in Scotland (*rescty*=S), Wales (*rescty=*W), Northern Ireland (rescty=X) or foreign (*rescty=*X).

**References**

1. Pan H, Cole T. LMSgrowth, a Microsoft Excel add-in to access growth references based on the LMS method. [Internet]. 2012 [cited 2019 Nov 15]. Available from: https://www.healthforallchildren.com/shop-base/shop/software/lmsgrowth/

2. Hardelid P, Dattani N, Davey J, Pribramska I, Gilbert R. Overview of child deaths in the four UK countries. 2013 [Internet]. London; 2013 [cited 2014 Nov 18]. Available from: http://scholar.google.com/scholar?hl=en&btnG=Search&q=intitle:Overview+of+child+deaths+in+the+four+UK+countries#7

3. NHS Digital. A Guide to Linked Mortality Data from Hospital Episode Statistics and the Office for National Statistics. 2015;(June). Available from: https://digital.nhs.uk/data-and-information/data-tools-and-services/data-services/linked-hes-ons-mortality-data#ons-mortality-data
