# Supplementary material for: Developing a national birth cohort for child health research using a hospital admissions database in England: The impact of changes to data collection practices
Source: PLoS One. 2020 Dec 15;15(12):e0243843. doi: 10.1371/journal.pone.0243843 (PMC7737962; doi:10.1371/journal.pone.0243843)
Supplement: S3 Appendix — (DOCX) [file pone.0243843.s003.docx]

# S3 Appendix– additional results

## Interrupted time series model

S3 Table A – Comparison of trends (change in intercept and slope) in the proportion of children with any hospital readmissions after birth in the first year of life from interrupted time series analysis

|  | all children | | Children with short birth admission (0-6 days) | Children with long birth admission (≥7 days) |
| --- | --- | --- | --- | --- |
| Proportion of children with any readmission in 1^st^ year of life in Q1 1998 (i.e. the intercept) | 13.5% (13.1%, 13.9%) | | 12.6% (12.2%, 13.0%) | 22.7% (22.1%, 23.4%) |
| Absolute annual change in the proportion of children with any readmission in 1^st^ year of life (change in slope): | | | | |
| Q1 1998-Q2 2002 | -0.2% (-0.2%, -0.1%) | | -0.2% (-0.2%, -0.1%) | -0.2% (-0.3%, 0.0%) |
| Q4 2002-Q2 2009 | 0.1% (0.1%, 0.2%) | | 0.1% (0.1%, 0.2%) | 0.2% (0.2%, 0.3%) |
| Q3 2009-Q1 2013 | 0.1% (0.1%, 0.1%) | | 0.1% (0.1%, 0.2%) | 0.1% (0.0%, 0.1%) |
| Q2 2013 onwards | 0.2% (0.1%, 0.3%) | | 0.2% (0.1%, 0.3%) | 0.1% (0.0%, 0.2%) |
| Change in the proportion of children with any readmission in the 1^st^ year of life (compared to expected value based on trend before, i.e. change in intercept) in: | | | | |
| Q4 2002 | 6.1% (4.8%, 7.3%) | | 5.9% (4.7%, 7.1%) | 8.6% (6.8%, 10.4%) |
| Q3 2009 | 0.5% (-0.2%, 1.1%) | | 0.5% (-0.2%, 1.1%) | 0.5% (-0.3%, 1.3%) |
| Q2 2013 | -0.3% (-1.1%, 0.4%) | | -0.3% (-1.1%, 0.4%) | -0.6% (-1.5%, 0.3%) |
| Seasonal increase in the proportion by quarter of year | |  |  |  |
| Q2 vs Q1 (across all years) | 1.4% (1.0%, 1.9%) | | 1.5% (1.0%, 1.9%) | 1.6% (1.0%, 2.1%) |
| Q3 vs Q1 (across all years) | 1.5% (1.1%, 1.9%) | | 1.5% (1.1%, 1.9%) | 2.0% (1.4%, 2.6%) |
| Q4 vs Q1 (across all years) | 1.2% (0.7%, 1.7%) | | 1.2% (0.7%, 1.7%) | 1.3% (0.7%, 2.0%) |
